# *Rdh10* loss-of-function and perturbed retinoid signaling underlies the etiology of choanal atresia

**DOI:** 10.1093/hmg/ddx031

**Published:** 2017-02-07

**Authors:** Hiroshi Kurosaka, Qi Wang, Lisa Sandell, Takashi Yamashiro, Paul A. Trainor

**Affiliations:** 1 Department of Orthodontics and Dentofacial Orthopedics, Graduate School of Dentistry, Osaka University, Osaka, Japan; 2 Department of Oral Immunology and Infectious Diseases, University of Louisville, School of Dentistry, Louisville, KY, USA; 3 Stowers Institute for Medical Research, Kansas City, MO, USA and; 4 Department of Anatomy and Cell Biology, University of Kansas Medical Center, Kansas City, KS, USA

## Abstract

Craniofacial development is a complex process that involves sequential growth and fusion of the facial prominences. When these processes fail, congenital craniofacial anomalies can occur. For example, choanal atresia (CA) is a congenital craniofacial anomaly in which the connection between the nasal airway and nasopharynx is completely blocked. CA occurs in approximately 1/5000 live births and is a frequent component of congenital disorders such as CHARGE, Treacher Collins, Crouzon and Pfeiffer syndromes. However, the detailed cellular and molecular mechanisms underpinning the etiology and pathogenesis of CA remain elusive. In this study, we discovered that mice with mutations in *retinol dehydrogenase 10 (Rdh10)*, which perturbs Vitamin A metabolism and retinoid signaling, exhibit fully penetrant CA. Interestingly, we demonstrate *Rdh10* is specifically required in non-neural crest cells prior to E10.5 for proper choanae formation, and that in the absence of *Rdh10*, *Fgf8* is ectopically expressed in the nasal fin. Furthermore, we found that defects in choanae development are associated with decreased cell proliferation and increased cell death in the epithelium of the developing nasal cavity, which retards invagination of the nasal cavity, and thus appears to contribute to the pathogenesis of CA. Taken together, our findings demonstrate that RDH10 is essential during the early stages of facial morphogenesis for the formation of a functional nasal airway, and furthermore establish *Rdh10* mutant mice as an important model system to study CA.

## Introduction

Craniofacial malformations comprise one of the most frequent classes of congenital anomalies in live human births (1). The high incidence is attributable to the complexity of craniofacial development, which depends on multiple precise biological events, including growth and fusion of the facial prominences and pharyngeal arches (2,3). Perturbation of these events can result in a wide variety of congenital craniofacial deformities, such as cleft lip and/or palate (4). Choanal atresia (CA), which is characterized by complete blockage of the connection between the nasal airway and nasopharynx (5), occurs in 1/5000–7000 human live births, and requires immediate airway management to prevent respiratory problems (6). CA is often a frequent feature of complex craniofacial disorders including CHARGE, Treacher Collins, Pfeiffer and Crouzon syndromes (7–9).

A number of mechanisms (10) have been hypothesized to underlie the pathogenesis of CA including (i) persistent buccopharyngeal membrane from the forgut; (ii), abnormal persistence or location of mesoderm forming adhesions in the nasochoanal region; (iii), abnormal persistence of the nasobuccal membrane of Hochstetter; and (iv), misdirection of neural crest cell migration (11–14). However, the detailed cellular and molecular mechanisms underpinning the etiology and pathogenesis of CA remain to be elucidated. In particular, we still do not know the critical period or tissues that are responsible for the pathogenesis of CA and a lack of relevant model for studying this condition contributes to our lack of knowledge.

In an ENU mutagenesis screen, which was aimed at identifying novel alleles that played critical roles in murine craniofacial development, we identified a mutant called *trex* which exhibited severe craniofacial anomalies including a midline facial cleft in association with mid-gestational lethality (15,16). We determined that the *trex* mice carried a mutation in *retinol dehydrogenase 10 (Rdh10)* and showed that RDH10 is responsible for oxidizing Vitamin A (retinol) to retinal (15), which is a rate-limiting step in the synthesis of retinoic acid (17,18). Commensurate with these discoveries, we demonstrated that *Rdh10^trex/trex^* mutant embryos exhibit deficiencies in retinoid signaling in association with their specific developmental anomalies (15,19–22), and furthermore that supplementation with the Vitamin A metabolic intermediate retinal, enabled *Rdh10^trex/trex^* mutant embryos to survive to birth (17). However, despite the remarkable overall prevention of their developmental anomalies, newborn *Rdh10^trex/trex^* pups often died shortly after birth, possibly as a result of a defect in oronasal development such as CA.

In order to investigate the precise role of *Rdh10* and retinoid signaling in oronasal development and in the etiology and pathogenesis of CA, we utilized an allelic series of *Rdh10* mutant mice including, *Rdh10^trex/trex^* and an *Rdh10* conditional knockout line (*Rdh10^flox^*) (18) crossed with a tamoxifen-inducible ubiquitous *Cre* mouse (23) so that *Rdh10* could be knocked out in a stage-specific manner (*Cre-ER^T2^; Rdh10^flox/flox^*) or tissue specific manner (*Wnt1-Cre;Rdh10^flox/flox^*) during embryogenesis. In the present study, we discovered that when tamoxifen was administered at E7.5 to induce excision of *Rdh10* by E8.5, *Cre-ER^T2^; Rdh10^flox/flox^* mutant embryos exhibited CA mimicking the phenotype observed in humans, including an obstructed airway with ectopic bone formation in the maxillary region. Mechanistically, we demonstrate that the expression of *Rdh10* in non-neural crest cells prior to E10.5 is critical for connecting the nasal airway and nasopharynx during normal choanae development. Furthermore, we show that *Fgf8* is ectopically expressed in the nasal epithelium in association with elevated cell death and reduced cell proliferation in *Rdh10* mutants, which perturbs morphogenesis of the nasal cavity, underpinning the pathogenesis of CA. Collectively, our findings reveal novel genetic pathways and cellular mechanisms underlying the etiology and pathogenesis of CA.

## Results

### *Rdh10* expression and retinoid signaling are present in the developing nasal cavity

**Figure 1:**
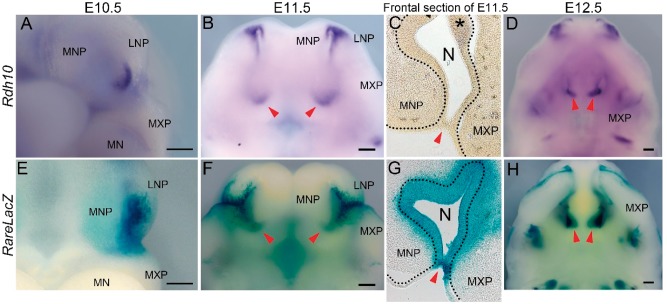
Expression of *Rdh10* and activity of *RARE-LacZ* during craniofacial development. (**A,B,D**) Ventral view of *Rdh10* expression in serial embryonic stages in wild-type mice. (**E**,**F,H**) Activity of *RARE-LacZ* reporter during embryonic maxillary development. Red arrowheads indicate the position of primitive choanae in B and F, invaginating nasal cavity in D and H. (**C** and **G**) Frontal section of E11.5 control embryo showing expression of *Rdh10* (C) and activity of *RARE-LacZ* (G). Red arrowhead indicates oronasal membrane in C and G, and black dotted line indicates the boundary of epithelial cells lining the nasal cavity. MNP, medial nasal process. LNP, lateral nasal process. MXP, maxillary process. MN, mandibular process. Scale bar = 200 μm.

As a first step in investigating the potential role for *Rdh10* and retinoid signaling in oronasal development and in the etiology of CA, it was important to define the spatiotemporal activity of *Rdh10* and retinoid signaling during early craniofacial development. Therefore, we performed *in situ* hybridization using a riboprobe for *Rdh10* in concert with *β-galactosidase* staining of retinoic acid response element (*RARE*) *-LacZ* reporter mice to document the distribution of retinoid signaling during embryogenesis (24). In E10.5 embryos, *Rdh10* mRNA was detected in the distal and lateral epithelium of the developing nasal pit (Fig. 1A), while an overlapping but somewhat broader domain of activity was observed with *RARE-lacZ* (Fig. 1E). As the primitive choanae begin to form around E11.5, *Rdh10* became more broadly expressed proximo-distally throughout the nasal epithelium in a pattern that was again overlapped by *RARE-LacZ* activity (Fig. 1B and F, red arrowhead). Histological sections confirmed that expression of *Rdh10* was largely confined to the nasal epithelia (Fig. 1C, dotted line, asterisk), whereas the domain of *RARE-LacZ* occupied both the epithelium, which includes the oronasal membrane (Fig. 1G, red arrowhead), and the adjacent mesenchyme of the medial nasal and maxillary prominences. As the nasal cavity continued to develop through E12.5, strong expression of *Rdh10* and staining for *RARE-LacZ* continued in both the proximal (red arrowheads) and distal tissues (Fig. 1D and H). Collectively, these data place *Rdh10* and retinoid signaling in the precise location at the appropriate time to play a prominent role in oronasal development and choanae formation.

### Efficient deletion of *Rdh10* activity in conditional knockout mice

**Figure 2:**
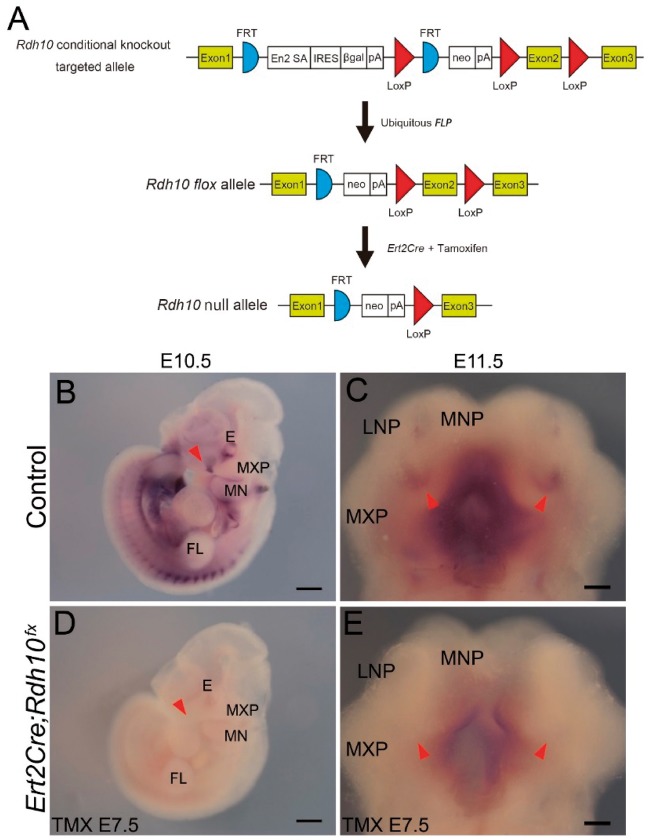
Confirmation of reduction of *Rdh10* mRNA in mice with conditional null allele. (**A**) Schematic drawing of the method used to generate the *Rdh10* conditional null allele (modified from reference (18). (**B–E**) *In situ* hybridization of *Rdh10* using an Exon2 specific riboprobe. E10.5 and E11.5 control embryos (B and C) compared with the same stage of *Cre-ER^T2^; Rdh10^flox/flox^* embryos (D and E) which were administered tamoxifen at E7.5. Red arrowhead indicates the lambdoidal region in B,D; and primitive choanae in C,E. TMX E7.5, tamoxifen was administered at E7.5. E, eye. MXP, maxillary process. MN, mandibular process. MNP, medial nasal process. LNP, lateral nasal process. FL, forelimb. Scale bar = 500 μm in B,D and 200 μm in C,E.

In order to demonstrate a functional requirement for *Rdh10* and retinoid signaling during oronasal development and in the etiology of CA, we temporally deleted *Rdh10* throughout the entire embryo during early stages of development (18). We crossed *Rdh10^flox/flox^* mice to *Cre-ER^T2^* mice (*Cre-ER^T2^; Rdh10^flox/flox^*) and administered tamoxifen to pregnant dams at E7.5 (Fig. 2A). First, we confirmed the efficiency of this conditional knock out allele by performing *in situ* hybridization using an Exon2 specific riboprobe for *Rdh10*. In wild type embryos at E10.5, *Rdh10* is expressed in a tissue-specific manner in the nasal pit amongst other tissues (Fig. 2B). In contrast, *Cre-ER^T2^; Rdh10^flox/flox^* mutant embryos generated by exposure to tamoxifen at E7.5 exhibited a substantial reduction of *Rdh10* expression throughout the entire body as expected and particularly in the nasal region (Fig. 2D). This phenomenon is similar to the loss of *Rdh10* expression previously reported in *Rdh10^trex/trex^* mutant embryos (15). Furthermore, the expression of *Rdh10* in the choanal region was considerably reduced in E11.5 *Cre-ER^T2^; Rdh10^flox/flox^* embryos when tamoxifen was administered at E7.5, compared to control littermates (Fig. 2C and E). Collectively, this demonstrates the efficient temporal generation of an *Rdh10* loss-of-function model which together with *Rdh10^trex/trex^* mice provides an allelic series for studying the role of *Rdh10* and retinoid signaling in oronasal development and the pathogenesis of CA.

### *Rdh10* loss-of-function results in CA with ectopic bone formation during craniofacial development

**Figure 3:**
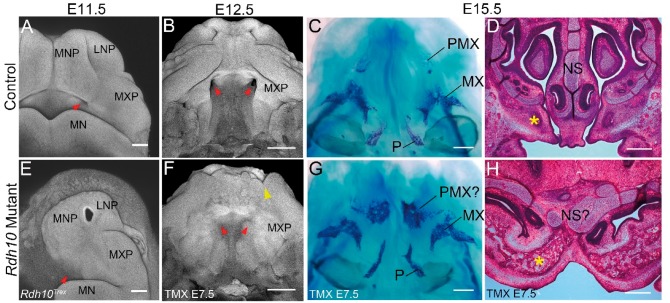
Morphological and histological analysis of *Rdh10* mutant maxilla. Nuclear fluorescent staining of control (**A,B**) and *Rdh10^trex^* (**E**), and *Cre-ER^T2^; Rdh10^flox/flox^* mice to which tamoxifen was administered at E7.5 (**F**). Red arrowhead indicates the position of primitive choana(e) in (A and B), and their absence (E and F). Yellow arrowhead in (F) shows partial cleft lip. Skeletal preparation of E15.5 control (**C**) and *Cre-ER^T2^; Rdh10^flox/flox^* embryos (**G**). Hematoxylin and eosin staining of frontal section of E15.5 control (**D**) and *Cre-ER^T2^; Rdh10^flox/flox^* (**H**) embryos. Yellow asterisk demarcates the premaxillary bone. TMX E7.5, administered tamoxifen at E7.5. MNP, medial nasal process. LNP, lateral nasal process. MXP, maxillary process. MN, mandibular process. PMX, premaxillary bone. MX, maxillary bone. P, palatine bone. NS, nasal septum. Scale bar = 200 μm in A,E and 500 μm in B-D, F-H.

The effect of *Rdh10* loss-of-function was initially determined morphologically via whole mount nuclear fluorescent (pseudo-SEM) imaging (25). In E11.5 control embryos, the primitive choanae starts to develop from the posterior region of the medial nasal process and maxillary process, as indicated by an epithelial invagination (Fig. 3A, red arrowhead). *Rdh10^trex^* embryos, which survive until around this stage without retinal supplementation, exhibit an absence of epithelial invagination, indicating that choanal development was impaired (Fig. 3E, red arrowhead). In E12.5, control embryos, oronasal development has progressed to the extent that the connection of the nasal cavity to the airway is complete (Fig. 3B, red arrowhead). In contrast, E12.5 *Cre-ER^T2^; Rdh10^flox/flox^* embryos, which were administered tamoxifen at E7.5, exhibit bilateral CA (Fig. 3F, red arrowhead) together with other defects such as cleft lip (Fig. 3F, yellow arrowhead). Alizarin red and alcian blue staining of the craniofacial skeleton of E15.5 *Cre-ER^T2^; Rdh10^flox/flox^* embryos revealed ectopic bone formation in the maxillary region (Fig. 3C and G). Frontal histological sections also revealed a substantially enlarged bone mass in the anterior maxillary region of E15.5 *Cre-ER^T2^; Rdh10^flox/flox^* embryos (Fig. 3H, asterisk) compared to controls (Fig. 3D, asterisk). In addition, the nasal airway failed to connect to the nasopharynx, and the shape of the nasal septum was severely distorted in *Cre-ER^T2^;Rdh10^flox/flox^* embryos compared to wild-type littermates (Fig. 3D and H). Taken together, these data demonstrate that *Rdh10* plays a critical role in oronasal development and that *Rdh10* loss-of-function can underpin the pathogenesis of CA.

### *Rdh10* loss-of-function results in ectopic *Fgf8* expression, reduced epithelial cell proliferation and elevated cell death

**Figure 4:**
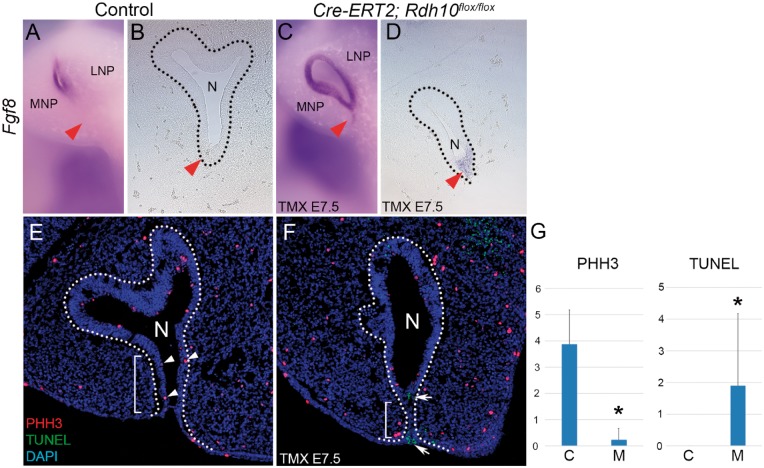
Excision of *Rdh10* resulted in elevation of *Fgf8* expression and altered cell proliferation and cell death. (**A–D**) *In situ* hybridization for *Fgf8* in E11.5 embryos. (B and D) Frontal section of E11.5 embryos hybridized with an *Fgf8* riboprobe. Red arrowheads indicate the nasal fin. (**E** and **F**) Immunohistochemistry of frontal sections from E11.5 embryos. Proliferating cells are stained red and cells undergoing cell death are stained green (E and F). White arrowheads indicate proliferating cells and white arrows indicate TUNEL-positive cell death in the nasal fin. Dotted line indicates the extent of the nasal epithelium. (G) Statistical analysis showed significant reduction in number of proliferating cell and elevation of cell death in *Cre-ER^T2^; Rdh10^flox/flox^* nasal fin. TMX E7.5, administered tamoxifen at E7.5. MNP, medial nasal process. LNP, lateral nasal process. N, nasal cavity. PHH3, phosphohistone H3. C, control mice. M, mutant (*Cre-ER^T2^; Rdh10^flox/flox^*) mice. *P < 0.05.

To better understand the molecular mechanisms by which diminished *Rdh10* causes CA, we performed comprehensive comparative transcriptome analyses of the developing maxillary complex in E11.5 wild-type and mutant *Cre-ER^T2^;Rdh10^flox/flox^* embryos via RNAseq. We initially focused on the *Fgf* signaling pathway because it is critical for craniofacial development and also known to associate with the etiology of CA (7,26). Among the *Fgf* signaling related genes expressed in the developing facial area (27), *Fgf8* showed the most significant difference between control and *Cre-ER^T2^;Rdh10^flox/flox^* embryos that were treated with tamoxifen at E7.5 (Supplementary Material, Table S1). To validate any spatiotemporal difference in the pattern of *Fgf8* expression between control and *Cre-ER^T2^; Rdh10^flox/flox^* mice, we performed *in situ* hybridization for *Fgf8* in the maxillary complex of E11.5 wild-type and mutant embryos. In control E11.5 embryos, *Fgf8* was not detected in the nasal fin, which is the thin mass of tissue separating the medial nasal and maxillary prominences (Fig. 4A and B, red arrowhead). In contrast, E11.5 *Cre-ER^T2^;Rdh10^flox/flox^* embryos, which were administered tamoxifen at E7.5, exhibited ectopic *Fgf8* expression in the nasal fin (Fig. 4C and D, red arrowhead). Since FGF8 signaling is known to influence cellular activities such as cell proliferation and cell death during embryonic craniofacial development (28,29), we performed immunostaining with phosphohistone H3 (PHH3), to assess for alterations in cell proliferation, and TUNEL staining to detect any changes in apoptosis. PHH3 immunostaining revealed a significant reduction in proliferating epithelial cells in the nasal fin of mutant embryos as compared to controls. Furthermore, the region of diminished proliferation coincided with the domain of ectopic *Fgf8* expression (Fig. 4E–G and E white arrowhead). At the same time, TUNEL staining revealed a significant elevation of cell death in the corresponding region in mutant embryos when compared to controls (Fig. 4E–G and F white arrow). It is well established that cell proliferation and cell death play critical roles during embryonic morphogenesis. Thus ectopic FGF8 signaling in association with elevated apoptosis and diminished proliferation in the nasal epithelium may contribute to malformation of the nasal cavity which appears to underlie the pathogenesis of CA. In addition to elevated expression of *Fgf8*, we discovered other genes that exhibited significant expression level changes in *Cre-ER^T2^; Rdh10^flox/flox^* mutant embryos. *Distal-less* gene family members *Dlx1* and *Dlx2* (Supplementary Material, Table S1), which are known to play important roles in craniofacial development (30), were also elevated.

### Spatiotemporal requirement for *Rdh10* for primitive choanae formation

**Figure 5:**
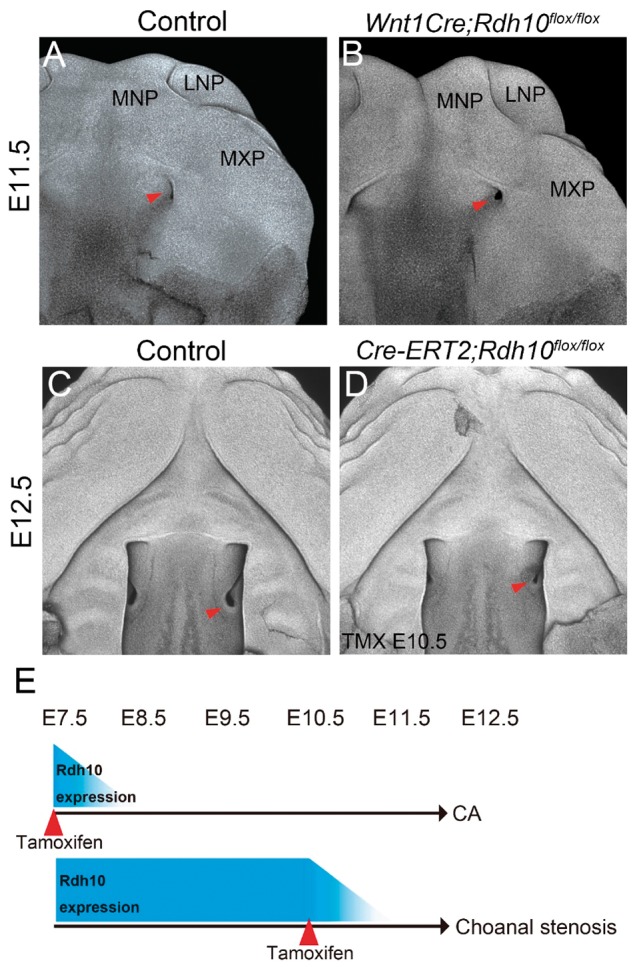
Morphological analysis following conditional deletion of *Rdh10* during craniofacial development. Nuclear fluorescent image of the maxillary complex in E11.5 control mouse embryos (**A**). Red arrowhead indicates the position of the primitive choana, which still forms in *Wnt1Cre;Rdh10^flox/flox^* (neural crest-specific deletion of *Rdh10*) mice (**B**). At E12.5, the nasal cavity invaginates posteriorly in control mice (**C**, red arrowhead), but is impaired and hypoplastic in E10.5 tamoxifen (TMX)-administered embryos (**D**, red arrowhead). (**E**) Schematic drawing of the phenotype of *Cre-ER^T2^; Rdh10^flox/flox^* embryos at E12.5 according to when the tamoxifen is administrated. TMX E10.5, administered tamoxifen at E10.5. MNP, medial nasal process. LNP, lateral nasal process. MXP, maxillary process.

Neural crest cells play critical roles in regulating craniofacial morphogenesis and it was important to determine whether *Rdh10* was intrinsically or extrinsically required in neural crest cells for normal oronasal development. Therefore, we used *Wnt1-Cre* mice (Supplementary Material, Fig. S1) to conditionally delete *Rdh10* from presumptive neural crest cells and their derivatives during embryogenesis. Nuclear fluorescent staining (pseudo-SEM imaging) clearly revealed the primitive choanae formation in both E11.5 control and *Wnt1Cre;Rdh10^flox/flox^* mutant embryos (Fig. 5A and B). This indicates that *Rdh10* is not required in neural crest cells for proper primitive choanae development. Next we determined the precise developmental period in which *Rdh10* was required for primitive choanae formation. E12.5 *Cre-ER^T2^;Rdh10^flox/flox^* embryos that were administered tamoxifen at E10.5 exhibited narrower choanae formation when compared to littermate controls which resembles choanal stenosis in humans (Fig. 5C and D, red arrowhead). Nonetheless, examination of sections of E13.5 wild-type and mutant embryos revealed that a connection does indeed form between the nasal airway and nasopharynx in *Cre-ER^T2^;Rdh10^flox/flox^* embryos (Supplementary Material, Fig. S2). Taken together, these results demonstrate that the formation of connection between the nasal airway and nasopharynx prior to E10.5. After E10.5 *Rdh10* is not needed for airway connection, but remains important for continued choanal development (Fig. 5E).

### Retinal supplementation ameliorates the pathogenesis of CA in *Rdh10* deficient mice

**Figure 6:**
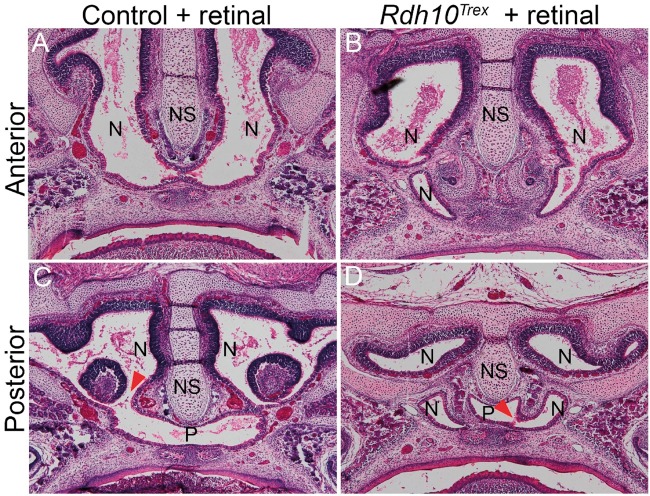
All-trans retinal rescued CA in *Rdh10^trex/trex^* mutants. (**A**) Hematoxylin and eosin staining of frontal section of anterior position of E18.5 control mouse and (**B**) *Rdh10^trex^* mutant treated with retinal. At the posterior position, the nasal cavity and pharynx are clearly connected in the control (**C**, red arrowhead) and in the *Rdh10^trex^* mutant treated with retinal (**D**, red arrowhead).

We have previously shown that the malformations observed in *Rdh10^trex/trex^* embryos can be largely rescued through maternal supplementation with retinal or retinoic acid during early embryogenesis (15,17,31,32). However, detailed examination of choana formation in embryos developing with retinal supplementation has not been performed. In order to characterize which aspects of choana development can be rescued by maternal retinal supplementation, we administered 12.5 mg/kg of retinal by oral gavage to *Rdh10^trex/+ ^* pregnant mothers, twice daily between E7.5 and E11, and subsequently harvested *Rdh10^trex/trex^* embryos and littermate controls at E18.5. Examination of frontal sections through the nasal septum and nasal cavity of wild-type and mutant embryos revealed differences in the shape of the nasal cavity. The nasal cavity in *Rdh10^trex/trex^* embryos was slightly wider and shorter than in littermate controls (Fig. 6A and B). However, from serial sections we observed that there was indeed complete penetration of the nasal cavity to the airway in retinal supplemented *Rdh10^trex/trex^* mice (Fig. 6C and D). When compared to the phenotype of non-supplemented *Rdh10^trex/trex^* embryos, as well as *Cre-ER^T2^;Rdh10^flox/flox^* embryos treated with tamoxifen at E7.5, this indicates that choanal defects in *Rdh10* mutant embryos result from insufficient production of retinal and addition of retinal by maternal administration is sufficient to promote connection of the airway.

## Discussion

### *Rdh10* and retinoid signaling play critical roles in the developing maxillary complex

It is well established that retinoid signaling plays an indispensable role in the development and patterning of various tissues and organs including craniofacial structures during embryogenesis (33–35). Retinoic acid is the metabolic byproduct of vitamin A generated by the two-step oxidation process. Excessive maternal intake of vitamin A is known to cause a significant elevation of retinoid signaling during embryogenesis, which results in craniofacial defects such as facial clefting in mammals and avians (36,37). *Rdh10* represents a nodal point in the feedback regulation of retinoid signaling and is thus a rate limiting factor in the first oxidation reaction which converts vitamin A (retinol) to retinal (15,17,18). Retinoic acid can either activate or repress the transcription of downstream target genes by binding to nuclear retinoic acid receptors (*RARs*), which in turn directly bind to specific DNA sequences called *RAREs* (33,38). In this study, we characterized the spatiotemporal patterns of *Rdh10* expression and retinoid signaling (*RARE-lacZ*) during mouse embryonic craniofacial development from stage E10.5 onwards with a particular emphasis on the oronasal region. *In situ* hybridization in E10.5 embryos revealed that *Rdh10* is strongly expressed in the epithelial lining of the developing nasal cavity, and is encompassed by a broader domain of active retinoid signaling, as shown by *RARE-lacZ* activity. The overlapping domains of *Rdh10* expression and *RARE-lacZ* activity continued through E11.5 and were maintained in the maxillary region, particularly where the primitive choanae develop. *Rdh10* is predominantly expressed in epithelial cells (Fig. 1C), while *RARE-lacZ* expression was detected in both epithelial and mesenchymal cells (Fig. 1G), which indicates that while retinoic acid might be produced primarily in epithelial tissue, the surrounding tissues actively respond to retinoid signaling. The intense pattern of *RARE-lacZ* staining observed in histological sections of the oronasal membrane (Fig. 1G, red arrowhead), suggests that retinoid signaling may play an important role in connecting the nasal airway and nasopharynx.

### Excising *Rdh10* during maxillary complex morphogenesis in mice leads to a phenotype resembling CA in humans

Previous studies revealed that germline mutation of *Rdh10* results in severe developmental defects, including craniofacial abnormalities and embryonic lethality around E11.5 (15,31). Here, we employed an allelic series of *Rdh10* mutant mice including conditional knockouts to investigate the role of *Rdh10* during craniofacial development and particularly in oronasal development and the pathogenesis of CA. Tamoxifen administration at E7.5 in combination with *Cre-ER^T2^* (39) resulted in the specific temporal and global deletion of *Rdh10* during early embryogenesis (Fig. 2C and E). These *Cre-ER^T2^; Rdh10^flox/flox^* embryos survived longer than *Rdh10^trex/trex^* embryos allowing for an investigation of the role of *Rdh10* in craniofacial development after E11.5, during which nasal cavity invagination and the primitive choanae formation occurs. In E11.5 *Rdh10^trex/trex^* embryos, epithelial invagination and the primitive choanae formation fails to occur resulting in CA (Fig. 3A,B,E and F).

These results clearly demonstrate that *Rdh10* is critically required between E7.5 and E10.5 for proper development of the primitive choanae, which is an important morphological step in connecting the nasal airway and nasopharynx. Furthermore, our histological analyses revealed ectopic bone formation in this obstructed area. Interestingly, the bone which was enlarged in *Cre-ER^T2^; Rdh10^flox/flox^* embryos seems to be part of developing premaxilla bone, while pterygoid plates and vomer bone are the bones affected in human CA. However, given the similarity in primitive choanae development between mice and humans (40), the allelic series of *Rdh10* mutant mice described in this study still provide useful models for investigating the etiology and pathogenesis of CA in humans.

### Disruption of retinoid and FGF8 signaling underpins the etiology of CA

Relatively few studies have investigated the cellular and molecular mechanisms underpinning CA using animal models. However, in support of our findings of a potential link between retinoid signaling and CA, mutations in *Aldh1a3* also cause CA. *Aldh1a3* plays a critical role in the second oxidation step of Vitamin A metabolism by converting retinal into retinoic acid. Furthermore, *Aldh1a3* is expressed in the developing nasal placode (41) and *Aldh1a3* loss-of-function mouse mutants exhibit CA in association with diminished retinoid signaling (26).

Interestingly, the phenotype of *Cre-ER^T2^; Rdh10^flox/flox^* embryos that were administered tamoxifen at E7.5 exhibited a wider spectrum of craniofacial deformities, including cleft lip and/or palate (CLP) (Fig. 3F), than *Aldh1a3* mutant embryos. CA in humans is known to be often associated with other craniofacial abnormalities including facial clefts (7,42), and there are other animal models which exhibit CA in combination with a facial cleft (26,43). The fact that *Rdh10* mutants exhibit either a midfacial cleft (15) or cleft lip (Fig. 3F) in combination with CA suggests that retinoid signaling mediated by *Rdh10* may contribute to the etiology of facial clefts in association with CA.

To further investigate the molecular basis of CA we undertook RNA-seq transcriptional analyses and identified the Fibroblast growth factor (FGF*)* signaling pathway as being altered in the maxillary complex of *Rdh10* mutant embryos (Supplementary Material, Table S1). *Fgf8* exhibited the most significant difference in expression level between control and *Cre-ER^T2^;Rdh10^flox/flox^* embryos that were treated with tamoxifen at E7.5 (Supplementary Material, Table S1). More importantly, we determined that *Fgf8* was ectopically expressed in the nasal fin of tamoxifen-administered *Cre-ER^T2^; Rdh10^flox/flox^* embryos (Fig. 4C and D).

Similar to our results reported here of ectopic *Fgf8* expression in *Rdh10* mutants, persistent *Fgf8* expression has also been observed in the nasal fin of *Aldh1A3* mutant embryos (26). Thus, elevated *Fgf8* as a consequence of reduced retinoid production may constitute a common mechanism leading to the manifestation of CA. These results indicate that CA in *Rdh10* and *Aldh1A3* mutant embryos shares a similar molecular etiology that starts with reduced retinoid signaling, and is followed by ectopic or elevated FGF8 signaling specifically in the nasal fin. Our working model therefore is that retinoid signaling is necessary to repress the activity of *Fgf8* during normal oronasal development. The role of retinoic acid in repressing *Fgf8* in oronasal development is therefore similar to the molecular competition between retinoid and FGF8 signaling that occurs during somitogenesis and in limb development (19–21). Moreover, studies of chick craniofacial development have demonstrated the disruption of *Fgf8* expression that occurs under conditions of reduced retinoid signaling (44,45). Furthermore, syndromes such as Pfeiffer syndrome, Crouzon syndrome, Apert syndrome, Beare–Stevenson syndrome and Antley-Bixler syndrome (46–48) exhibit CA in association with other craniofacial anomalies, and each has been linked to exaggerated FGF signaling. Finally, a recent study revealed that elevated *Fgf8* expression in cranial neural crest cells results in perturbation of nasal cavity invagination (49). This provides additional evidence supporting our observations and conclusions of the importance of retinoid and FGF signaling integration in the etiology of CA in *Rdh10* mutants.

FGF8 signaling is well known to play critical roles during craniofacial development through interactions with other signaling pathways and by regulating cellular activities such as cell proliferation and survival (49,50). In the present study, we discovered reduced cell proliferation and increased cell death in the nasal fin epithelium of *Rdh10* mutant embryos in association with ectopic FGF8 signaling (Fig. 4).

**Figure 7:**
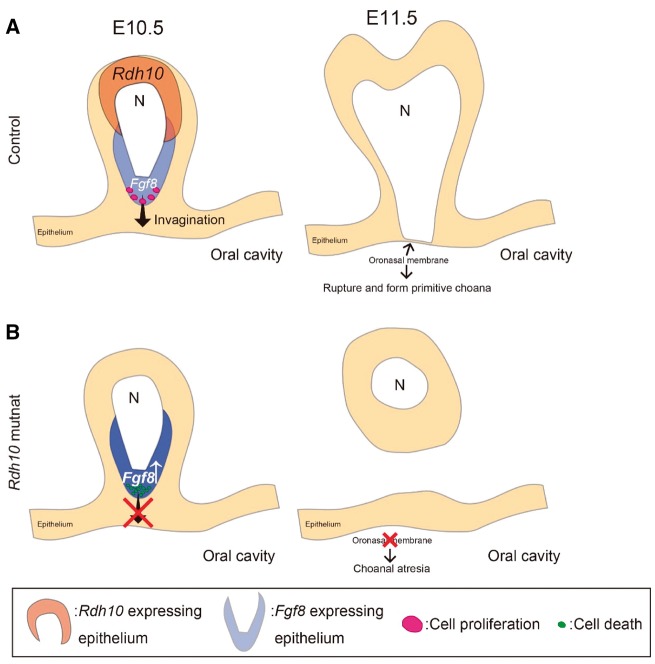
Schematic drawing of the process of nasal cavity invagination and primitive choanae formation. Nasal cavity invagination takes place to connect the nasal cavity to the airway by rupturing the oronasal membrane during normal development (**A**). In *Rdh10* mutants, the number of proliferating epithelial cells is reduced, and the nasal cavity cannot connect to the airway, resulting in CA (**B**).

Mitotic cell rounding is known to be associated with epithelial invagination (51,52) and thus it’s tempting to speculate that reduced proliferation in the nasal epithelium of *Cre-ER^T2^; Rdh10^flox/flox^* mutant embryos is associated with a reduction in mitotic cell rounding, resulting in impaired invagination and CA (Fig. 7). Furthermore, the increased cell death observed in *Cre-ER^T2^; Rdh10^flox/flox^* mutant embryos may actually then eliminate the cells that are important for invagination of the nasal cavity and primitive choanae development (Fig. 7).

In addition to elevated expression of *Fgf8*, we noted elevated expression of *Dlx1* and *Dlx2*, two established targets of FGF8 signaling (53,54). The observation that FGF8 target genes are elevated in addition to *Fgf8* strengthens the conclusion that ectopic FGF8 signaling plays an active role in abnormal facial morphogenesis in *Rdh10* mutant embryos. The possible roles or interaction of those genes with retinoid signaling during craniofacial development and in the pathogenesis of choanal atresia will be pursued in the future.

### *Rdh10* is spatiotemporally required for proper nasal cavity and primitive choanae development

Although we detected *Rdh10* expression in both the epithelium and mesenchyme of the developing nasal cavity, and know that crosstalk between epithelial and mesenchymal tissues are critical for normal organogenesis, it remained to be determined when and in which tissue *Rdh10* was critical required for proper primitive choanae formation. We found no obvious evidence for CA in embryos in which *Rdh10* was conditionally deleted in neural crest cells or temporally deleted throughout the embryo at E10.5. Collectively, these data strongly suggest that *Rdh10* is critically required between E7.5 and E10.5 in the non-neural crest cell derived nasal epithelium in order to connect the nasal airway and nasopharynx.

Mutant mice, which were administered tamoxifen at E10.5 exhibited narrower choanae which could mimic choanal stenosis in humans (Fig. 5D). This suggests that a common mechanism could underlie choanal atresia and choanal stenosis with choanal atresia being a more severe form of choanal stenosis in cases of disrupted retinoid signaling.

There is, however, clear evidence that defects in cranial neural crest cell development can also contribute to the etiology of CA as exemplified by the phenotype of Treacher-Collins syndrome which is classified as a neurocristopathy (2,9). These results imply that different signaling pathway may work in a tissue specific manner to promote the proper development of a functional airway.

Craniofacial morphogenesis is a complex process requiring growth and fusion of the medial and lateral nasal processes, together with nasal cavity invagination for proper oronasal development (55). Interestingly, the embryonic craniofacial development is a highly conserved process as has been shown through principal component analyses of comparative anatomy (56). This morphological conservation in combination with our results suggests that *Rdh10* is an important gene whose expression needs to be tightly regulated in a spatiotemporal manner for proper orofacial development.

### Maternal retinal supplementation could restore the CA phenotype in *Rdh10* mutant

Consistent with the idea of a precise spatiotemporal requirement for *Rdh10* during oronasal development, we showed that the pathogenesis of CA in *Rdh10^trex/trex^* mice could be prevented by administering retinal from E7.5 through E11.5. Taken together with the temporal deletion of *Rdh10* in *Cre-ER^T2^; Rdh10^flox/flox^* embryos following tamoxifen administration, these results again indicate that *Rdh10* and retinoid signaling is critically required between E7.5-E10.5 for choanae formation and for connecting the nasal airway and nasopharynx. Furthermore, this is consistent with our previous studies showing that maternal retinal or retinoic acid supplementation can rescue the gross morphology and embryonic lethality of *Rdh10* mutant embryos (15,31,32).

## Materials and Methods

### Animals

The *Rdh10^trex^* and *Rdh10^flox^* mice lines used in this study were generated and maintained as described previously (15,18). Briefly, the *Rdh10^flox^* mice were derived from ES cells generated through KOMP and is equivalent to C57BL/6N-Rdh10^tm1a(KOMP)Wtsi^*. Cre-ER^T2^* (B6.129-Gt(ROSA)26Sortm1(cre/ERT2)Tyj/J, Jax stock #008463), and *Wnt1Cre* mice (Tg(Wnt1-cre)11Rth Tg(Wnt1-GAL4)11Rth/J, Jax stock #003829) were obtained from the Jackson Laboratory and maintained as previously described (39,57,58). *Cre* driver mouse lines were crossed with *Rdh10^flox^* mice in order to eliminate RDH10 function in neural crest cells (*Wnt1Cre*) or in all embryonic cells following treatment with tamoxifen (*Cre-ER^T2^*). The homozygous *Rdh10* flox alleles in combination with either *Cre-ER^T2^* or *Wnt1Cre* are respectively termed *Cre-ER^T2^; Rdh10^flox/flox^* and *Wnt1Cre; Rdh10^flox/flox^* in this manuscript, and either wild-type or heterozygous littermates were used as controls. *RARE-LacZ* (Tg(RARE-Hspa1b/lacZ)12Jrt/J, Jax stock #008477) and *Rosa 26 Reporter* (*R26R*) (FVB.129S4(B6)-Gt(ROSA)26Sortm1Sor/J, Jax stock #009427) reporter mice were also obtained from The Jackson Laboratory and maintained as described previously (24,59). For embryonic staging, the morning of identification of the vaginal plug was defined as E0.5.

### Administration of tamoxifen

To activate the *Cre* protein at defined stages of embryonic development, we administered 5 mg of tamoxifen and 1 mg of progesterone in 100 μl of corn oil by oral gavage to individual pregnant female *Rdh10^fllox/flox^* mice at defined time points after they had been successfully mated with an *Ert2Cre:Rdh10 ^fllox/flox^* male.

### *In situ* hybridization

Whole-mount *in situ* hybridization and subsequent sectioning of stained embryos were performed as described previously (3). Exon2 of *Rdh10* was amplified using the following set of primers: forward 5' CTGGAAAAGGTGAGGAGGAAATC 3', reverse 5' CCAGAAGTGTGCGTGGCAGTTG 3', and cloned into the TOPO vector (Invitrogen) to produce an RNA riboprobe.

### Whole-mount nuclear fluorescent imaging

To analyze embryonic morphology, a nuclear fluorescent imaging technique called ‘Pseudo SEM’ was performed as previously described (25).

### Skeletal preparation

Embryos were collected at E16.5 and stained with Alizarin red and alcian blue to visualize bone and cartilage, respectively, as previously described (60).

### Immunohistochemistry and TUNEL staining

Embryos were dissected at various defined developmental stages, fixed in 4% PFA at 4 °C overnight, equilibrated through a sucrose gradient to 30%, and mounted in Tissue Tek O.C.T. 10-μm sections were cut, mounted on glass slides and stored at −30°c until required. Immunohistochemistry was performed with an M.O.M. Immunodetection Kit (VECTOR) according to the manufacturer’s protocol. After the sections were incubated with the primary antibody (anti-phospoho-Histone-3 (Millipore)), they were washed and incubated with a fluorescent secondary antibody and counterstained with DAPI (DAKO). To detect the presence of apoptosis, we used an *In Situ* Cell Death Detection Kit Fluorescein (Roche) according to the manufacturer’s instructions.

### *β-* *g* *alactosidase* staining

*β-Galactosidase* staining of *RARE-LacZ* and *Wnt1Cre;R26R* mouse embryos was performed as described previously (3). For detailed analysis, some of the stained embryos were processed into 18-μm-thick frozen sections.

### Maternal supplementation with retinal

*Rdh10^trex/+ ^* pregnant female mice that had been mated with *Rdh10^trex/+ ^* males were administered twice per day (morning and evening) by oral gavage, 12.5 mg/kg weight retinal from E7.5 through E11.5.

### RNAseq analysis

The maxillary complex (including medial nasal, lateral nasal and maxillary processes) (3) was isolated from E11.5 *Cre-ER^T2^; Rdh10^flox/flox^* mouse embryos (*n =* 3) and their littermate controls (*n =* 3) that had been treated with tamoxifen via oral gavage of their mothers at E7.5. Total RNA was extracted using an RNeasy Kit (Qiagen) according to the manufacturer’s protocol and RNA-seq was performed using 2 of each samples which showed a similar overall expression profile to analyze the data. Sequences of each RNA sample were aligned using TopHat (v2.0.10) (61). The reference genome and annotation files used for the alignment were from UCSC mm10 genome and ensemble build 75. We generated read counts that were mapped to the exons of genes using BEDTOOLs. Normalized gene expression (RPKMs) is calculated using the rpkm function after the TMM normalization with R package edgeR (62).

### Statistics

*P* values in Figure 4E were adjusted using the Benjamini-Hochberg procedure to control the false discovery rate. An R package, edgeR, was used to find differentially expressed genes between control and mutant samples. *t*-test that assumes non-equal variances were performed for data represented in Figure 4H. Adjusted *P*-values of less than 0.05 were considered significant in all experiments.

### Study approval

Mice were housed either in an animal facility in Department of Dentistry, Osaka University or the Laboratory Animal Services Facility at the Stowers Institute for Medical Research. Welfare guidelines and procedures were performed with the approval of Osaka University Graduate School of Dentistry animal committee and the Stowers Institute for Medical Research IACUC.

## Supplementary Material

Supplementary Material is available at *HMG* online.

## Supplementary Material

Supplementary Data
