# Supplementary material for: Rdh10 loss-of-function and perturbed retinoid signaling underlies the etiology of choanal atresia
Source: Hum Mol Genet. 2017 Feb 7;26(7):1268–79. doi: 10.1093/hmg/ddx031 (PMC5390677; doi:10.1093/hmg/ddx031)

**Supplemental data**

**Supplemental Table.1**

|  | Normalized gene expression (FPKM) | |  |
| --- | --- | --- | --- |
| Gene | Control | Rdh10 mutant | Adjusted P value (FDR) |
| ***Fgf8**** | 2.24 | 4.00**↑** | **2.64E-08** |
| ***Fgf10**** | 0.20 | 0.17**↓** | **0.04** |
| *Fgf9* | 0.23 | 0.25↑ | 0.71 |
| *Fgf15* | 0.46 | 0.38↓ | 0.57 |
| *Fgf17* | 1.28 | 1.66↑ | 0.11 |
| *Fgfr1* | 3.92 | 3.85↓ | 0.91 |
| *Fgfr2* | 0.30 | 0.30→ | 0.99 |
| ***Dlx1**** | 2.33 | 3.88**↑** | **0.0003** |
| ***Dlx2**** | 5.32 | 8.28**↑** | **1.01E-05** |

**Supplemental Figure.1**


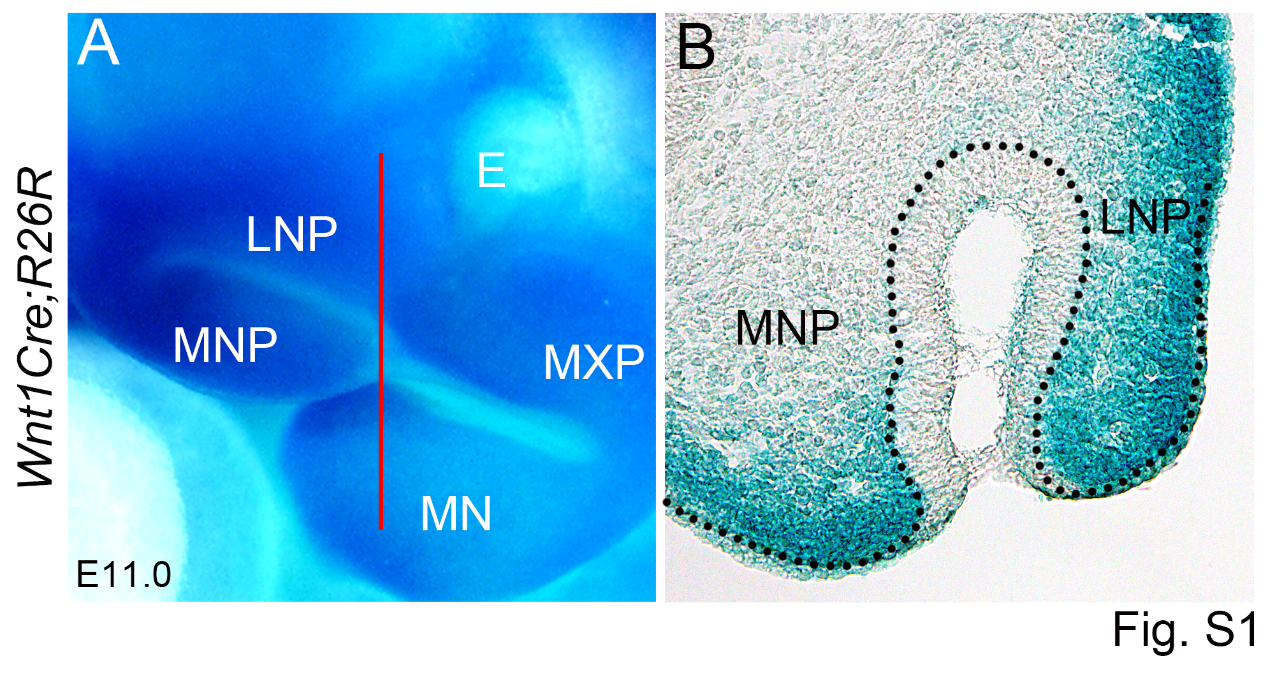


**Supplemental Figure.2**


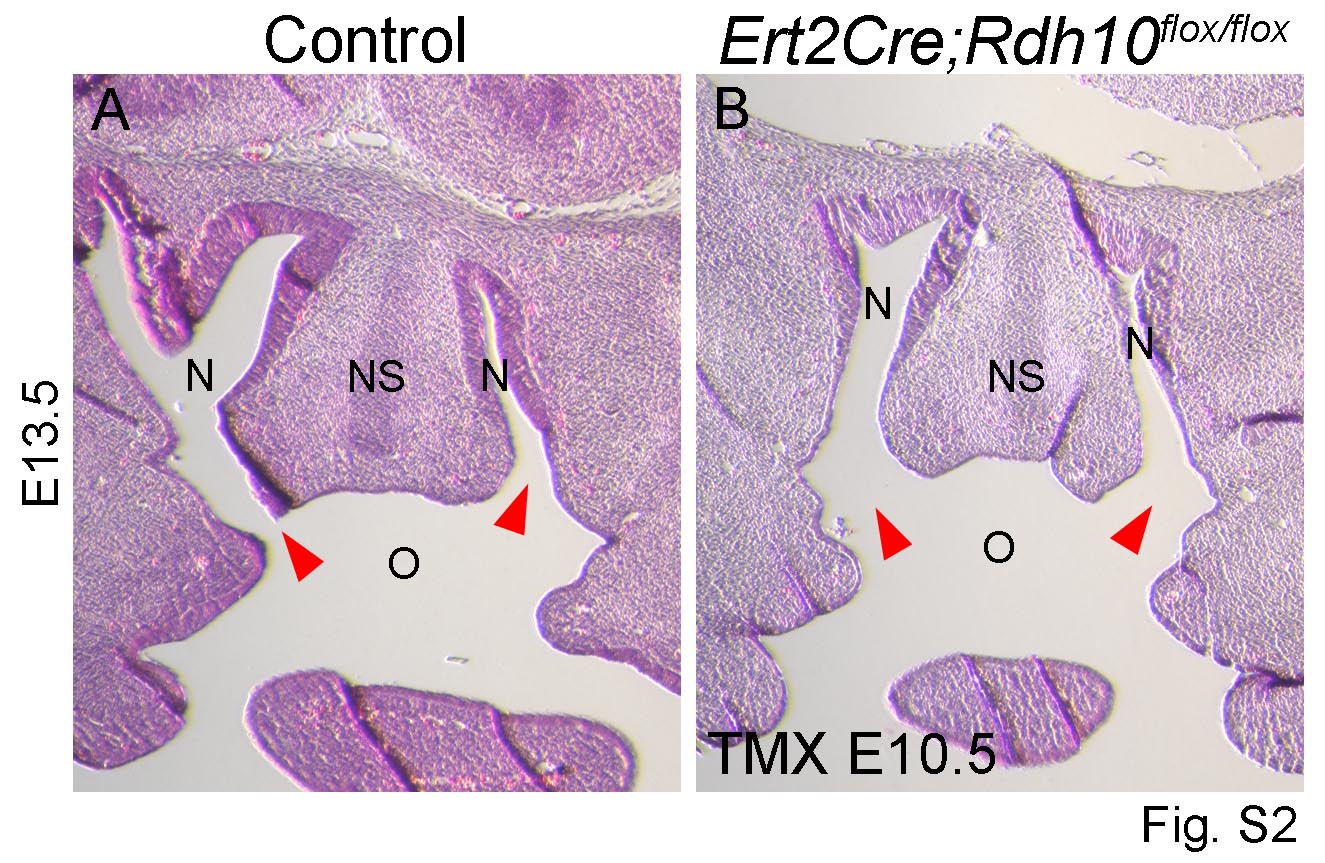

Supplement: Supplementary Data [file ddx031_Supp.docx]
